# MicroRNA-92 regulates cervical tumorigenesis and its expression is upregulated by human papillomavirus-16 E6 in cervical cancer cells

**DOI:** 10.3892/ol.2013.1404

**Published:** 2013-06-17

**Authors:** YU YU, YAO ZHANG, SHULAN ZHANG

**Affiliations:** Department of Obstetrics and Gynecology, Shengjing Hospital of China Medical University, Shenyang, Liaoning 110001, P.R. China

**Keywords:** squamous cervical carcinoma, microRNA -92, phosphatase and tensin homologue, human papillomavirus-16 E6

## Abstract

MicroRNA (miR)-92 is overexpressed in a number of tumors and has been proven to negatively regulate a number of tumor suppressor genes, including phosphatase and tensin homologue (PTEN). However, its function and molecular mechanism(s) of action in squamous cervical carcinoma (SCCs) have not been well described. Furthermore, the correlation between miR-92 and human papillomavirus (HPV)-16 E6 has not been studied. In the present study, miR-92 expression levels were quantified using quantitative PCR (qPCR) in cervical cancer tissues, normal cervical tissues and cervical cancer cell lines. SiHa cells were transfected with either miR-92-mimics, anti-miR-92 or negative controls. C33A cells were stably transfected with pEGFP-N1-16E6 and pEGFP-N1-neo plasmids. The levels of PTEN protein expression in the transfected SiHa and C33A cells were evaluated using western blot analysis. The effects of miR-92 were detected using cell counting kit (CCK)-8 and Transwell assays. HPV16 E6 siRNA was used to detect the effectiveness of the E6 protein on miR-92 in the SiHa and C33A cells. miR-92 was highly-expressed in the human cervical cancer tissues compared with the normal tissues. In the HPV16-positive cervical cancer tissues, the expression of miR-92 was higher compared with the HPV16-negative cervical cancer tissues. HPV16 E6 upregulated miR-92 expression in the SiHa- and C33A-pEGFP-N1-16E6 cells. The upregulation of miR-92 promoted cell growth and invasion in the SiHa cells. PTEN protein expression was decreased in the SiHa cells that were transfected with the miR-92 mimic. The data indicated that miR-92 may increase the migration and invasion of SiHa cells, partially through the downregulation of PTEN protein expression. HPV16 E6 was identified to upregulate miR-92 expression.

## Introduction

In developing countries, cervical cancer is a leading cause of mortality among females and is the second most common malignancy in females worldwide (1). Each year, >270,000 females succumb to cervical cancer, of which 53,000 are located in China (2). Studies have demonstrated that early sexual intercourse, promiscuity, infection with high-risk types of human papillomavirus (HPV) and a number of other factors may cause the normal cervical epithelium to become pre-neoplastic cervical intraepithelial neoplasia, which may later transform into invasive cervical cancer (2). However there has not been a viable explanation for the pathogenesis of cervical cancer.

MicroRNAs (miRs/miRNAs) are non-coding small RNAs composed of 19–25 ribonucleic acid molecules. They regulate the stability and expression of target mRNAs and serve as post-transcriptional regulators, which determine cell identity and fate. These small RNAs regulate gene expression by interacting with the 3′UTR of target mRNA (3–5). The expression levels of the members of the miR-17-92 cluster have been shown to be altered in numerous types of cancer. The miR-17-92 cluster encodes six miRNAs, miR-17, miR-18a, miR-19a, miR-19b, miR-20a and miR-92, which are located in a coding region of the open reading frame (ORF) of the C13orf25 gene (6–8). The human miR-17-92 cluster gene is mapped to chromosome 13q31. Data have indicated that the mir-17-92 cluster is involved in the regulation of cell growth, cell differentiation, apoptosis, cell motility, cell adhesion, cell invasion and angiogenesis (9,10). Previous evidence has shown that a number of miRNAs play significant roles in cancer by altering the expression of target oncogenes and tumor suppressor genes, including those affecting lung cancer, breast cancer, human glioma and lymphoproliferative disease (11–13). The phosphatase and tensin homologue (PTEN) protein is an important target protein. The significance of the PTEN protein is indicated by the fact that it is frequently disrupted in numerous cancers (14). TargetScan (http://www.targetscan.org/) has predicted hundreds of miR-92 targets and the tumor suppressor PTEN is one of the most prominent. PTEN has been shown to play a role in several human cancers (15,16). However, the correlation between miR-92 and PTEN has not previously been reported in cervical cancer. Whether miR-92 has any association with HPV remains undetermined. The present study aimed to examine miR-92 expression in HPV16-positive squamous cervical carcinomas (SCCs). The correlation between miR-92 expression and clinical status was also evaluated. miR-92-mimics and anti-miR-92 were transfected into cells from the SCC cell line, SiHa, and the contribution of miR-92 to tumor cell growth, apoptosis and migration was consequently investigated. The role of miR-92 in tumor formation was also evaluated using subcutaneously-inoculated immunocompromised mice. HPV16 E6 siRNA was transfected into SiHa cells and the pEGFP-N1-HPV16E6 plasmid and HPV16 E6 siRNA were transfected into C33A cells in order to detect the correlation between HPV16 E6 and miR-92. The roles of miR-92 and PTEN were investigated in cervical cancer cell lines and the introduction of miR-92 was analyzed with regard to PTEN protein and mRNA expression.

## Materials and methods

### Tissue collection

A total of 34 cervical cancer tissue samples, 23 of which were HPV16-positive, were obtained from patients (mean age, 43.1±6.0 years) by biopsy during colposcopy. Another 34 normal, HPV-negative cervical tissues were also collected from patients (mean age, 45.7±4.71 years) during hysterectomies. The samples were all obtained from the Shengjing Hospital of China Medical University (Shenyang, Liaoning, China). All the cervical cancer tissues were obtained from cervical squamous carcinomas that were confirmed by pathological analysis. All tissues were obtained with informed consent in accordance with the requirements of the China Medical University Research Ethics Committee, as stipulated in the Helsinki Declaration. This study was approved by the ethics committee of Shengjing Hospital of China Medical University.

### Cell lines and culture conditions

SiHa cells were obtained from the Shengjing Hospital of China Medical University. C33A cells were obtained from the American Type Culture Collection (ATCC, Manassas, VA, USA). The SiHa cells were cultured in Dulbecco’s modified Eagle’s medium (DMEM; Hyclone, Logan, UT, USA). The C33A cells were cultured in ATCC-formulated Eagle’s minimum essential medium (EMEM). The media were contained in 10% fetal bovine serum (FBS), 100 IU/ml penicillin and 100 mg/ml streptomycin. All cells were cultured at 37°C in a humidified atmosphere of 5% CO_2_.

### Transfection

The miR-92-mimic, anti-miR-92, negative control (NC), E6 siRNA and E6 siRNA-NC were purchased from Shanghai GenePharma Co., Ltd and Shanghai Sangon Co., Ltd, (Shanghai, China), and all contained green fluorescent tags. The SiHa cells were transfected with miR-92-mimic, anti-miR-92 and NC using Lipofectamine 2000 Reagent (Invitrogen, Carlsbad, CA, USA) according to the manufacturer’s instructions. The medium was replaced with fresh growth medium following 6 h of transfection. A qPCR analysis of miR-92 was used to detect the transfection efficiency.

pEGFP-N1-16E6 and pEGFP-N1-neo plasmids were stably transfected into the C33A cells. Briefly, the C33A cells were seeded in 24-well plates with 500 *μ*l EMEM medium and without antibiotics. The cells were transfected with 0.8 *μ*g pEGFP-N1-neo and pEGFP-N1-16E6 plasmids and selected in medium containing G418 (250 *μ*g G418 per ml). At two weeks post-transfection, the G418-resistant colonies were pooled and expanded.

### SYBR-green-based qPCR

Total RNA was extracted using the TRIzol reagent (Invitrogen). The cDNA was reverse-transcribed from 1 ng total RNA with a miRNA reverse transcription kit (SYBR Green-based Real Time PCR kit, Shanghai GenePharma Co., Ltd.). This cDNA was used at 3-fold dilutions for each run of qPCR on an ABI 7500 thermal-cycler system, according to the manufacturer’s instructions. The procedure was normalized using U6 as the endogenous control. The reverse transcription reaction was performed at 16°C for 30 min, 42°C for 30 min and 85°C for 10 min and then stored at 4°C. The SYBR Green qPCR reaction conditions consisted of an initial denaturation cycle at 95°C for 3 min, followed by 40 cycles at 95°C for 12 sec and annealing and extension at 62°C for 50 sec. Each experiment was repeated three times. All PCR products were resolved in agarose gel to confirm PCR specificity.

### Western blot analysis

The SiHa cells were transfected with miR-92-mimic and NC. At 48 h post-transfection, RIPA buffer (1 lg/ml leupeptin and 1 lg/ml PMSF; Beyotime, Nanjing, China) was used to isolate the total protein from the transfected cells in order to detect PTEN protein expression, in accordance with the manufacturer’s instructions. The proteins were separated using 10% sodium dodecylsulfate-polyacrylamide gel electrophoresis and transferred to polyvinylidene difluoride membranes. The membranes were blocked using 5% skimmed powdered milk for 2 h at room temperature. The membranes were washed with TBST and incubated with the primary antibody for PTEN (Santa Cruz Biotechnology, Inc., Santa Cruz, CA, USA), overnight at 4°C. The membranes were then washed using TBST and incubated with horseradish peroxidase (HRP)-labeled goat anti-mouse secondary antibodies (Beyotime). The bands were analyzed using the SuperSignal West Pico kit (Pierce, Rockford, IL, USA) and examined using ImageQuant enhanced chemiluminescence (ECL; GE Healthcare, Buckinghamshire, UK). The band intensities were analyzed using Quantity One software (Bio-Rad, Hercules, CA). Each experiment was repeated three times.

### Immunohistochemistry

The HPV16 E6 mouse polyclonal antibody was purchased from Santa Cruz Biotechnology, Inc. The cervical cancer tissue sections were deparaffinized and rehydrated through a series of graded alcohols and xylene, then washed with PBS (0.02 M). Endogenous peroxidase activity was blocked by 3% H_2_O_2_ for 15 min at room temperature, then exposure to the antigen. The tissue sections were incubated with the primary antibody for HPV16 E6 (HPV16 E6 antibody diluted to 1:50 and applied to each slide) overnight at 4°C. The tissue sections that were incubated with DAB (GSGB Biotechnology, Beijing, China) developed coloration. The HPV16 E6 protein was mainly expressed in the nucleus.

### Cell counting kit (CCK)-8 assay

Cell survival was assessed using a CCK-8 assay (Dojindo, Kumamoto, Japan). SiHa cells were transfected for 48 h in 96-well plates and incubated with CCK-8 for 24, 48, 72 and 96 h. Optical density was read at 450 nm (A450) using Easy Reader 340 AT (SLT-Lab Instruments, Bath, UK). Each experiment was repeated three times.

### Transwell cell migration assay

The SiHa cells were transfected with the miR-92-mimic and NC in 6-well plates. At 48 h post-transfection, the cells were collected and 5×10^4^ were placed in the upper chambers of the Transwell inserts with 5% FBS (8-*μ*m pore size; Corning Inc., Corning, NY, USA). The lower compartments contained DMEM medium with 20% FBS. Subsequent to 24 h, the cells that had migrated were fixed with 4% paraformaldehyde and crystal violet stain. The cells were then counted and images were captured.

### Caspase-3 activity

Caspase-3 activity was assessed using the colorimetric CaspACE Assay System (Promega, Mannheim, Germany), according to the manufacturer’s instructions. Briefly, equal amounts of cell extract were incubated with the substrate (Ac-DEVD-pNA) in the assay buffer for 24 h at room temperature. Absorbance was measured at 405 nm using a microplate reader (SLT-Lab Instruments). Each experiment was performed in triplicate.

### Terminal deoxynucleotidyl transferase-mediated dUTP nick end labeling (TUNEL) assay

The cells were washed in PBS supplemented in 0.1% bovine serum albumin and treated with an *in situ* detection kit, according to the manufacturer’s instructions (Boehringer Mannheim Biochemicals, Indianapolis, IN, USA). Nuclei with fragmented DNA were visualized using a fluorescence microscope.

### Tumor xenografts

Four-week-old female nude mice were cared for in accordance with the Guide for the Care and Use of Laboratory Animals (NIH publication no. 80-23, revised 1996), and the experiments were performed according to the Shengjing Hospital of China Medical University ethical guidelines for animal experiments. SiHa cells (1×10^6^) were transfected with anti-miR-92 or anti-miRNA-NC for 48 h. The cells were suspended in 200 *μ*l PBS and subcutaneously injected into the right and left posterior flanks of the same female BALB/c athymic nude mouse. A total of 20 nude mice were used in the experiment. Tumor growth was examined every third day for four weeks. The tumor volume (V) was calculated by measuring the tumor length (L) and width (W) using calipers and calculated using the following formula (L × W^2^) × 0.5. The tumor xenografts were harvested and snap-frozen. Cryosections (4 *μ*m) were stained using hemotoxylin and eosin.

### Statistics

All statistical analyses were carried out using SPSS for Windows, version 18.0 (SPSS, Inc., Chicago, IL, USA). All values are presented as the mean ± SD from at least three separate experiments. All tests that were performed were two-sided Student’s t-tests. P<0.05 was considered to indicate a statistically significant difference. Standard curves were generated and the relative amount of miR-92 expression was normalized to U6 snRNA. The miR-92 expression fold change was evaluated using the 2^−ΔΔCt^ method.

## Results

### miR-92 expression in cervical cancer tissues

**Figure 1.**
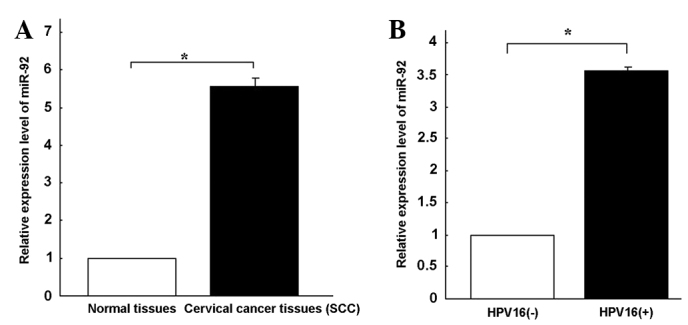
Differential expression of miR-92 in human cervical cancer tissues. (A) miR-92 expression in the 34 cervical cancer tissues compared with the 34 normal tissues detected using qPCR. U6 snRNA was used as an endogenous normalizer. (B) Relative expression of miR-92 in HPV16-positive cervical cancer tissues (^*^P<0.05). qPCR, quantitative PCR; miR-92, microRNA-92; HPV, human papillomavirus; SCC, squamous cervical cancer.

miR-92 expression was quantified in 34 tumor samples and 34 normal cervical tissues using qPCR (Fig. 1A). miRNA expression was relatively stable in the adjacent normal cervical tissues. miR-92 expression was 5.56-fold higher in the SCC tissues compared with the corresponding non-tumor tissues. These results suggest that miR-92 upregulation may play a role in the malignant progression of cervical cancer.

### Correlation between HPV16 infection and the upregulation of miR-92 expression in cervical cancer

**Figure 2.**
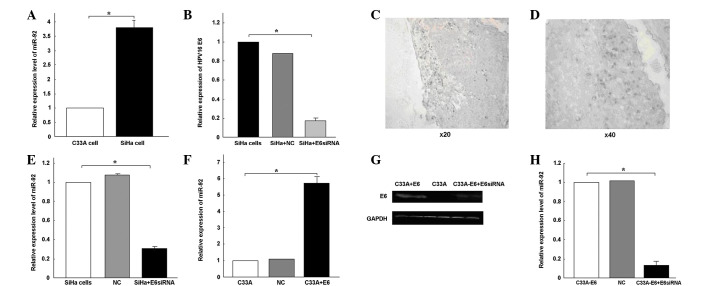
Correlation between miR-92 and HPV16 E6. (A) miR-92 expression compared with U6 snRNA in SiHa and C33A cells (^*^P<0.05). (B) E6 expression detected in SiHa cells that were transfected with HPV16 E6 siRNA (^*^P<0.05). (C and D) E6 oncoprotein expression detected in HPV16-positive tissues using immunohistochemistry [(C) ×200 magnification, and (D) ×400, magnification]. Subsequent to transfecting the cells with E6 siRNA, miR-92 expression levels were detected in (E) the SiHa cells (^*^P<0.05) and (F) the C33A cells (^*^P<0.05). (G) E6 oncoprotein expression was assessed in transfected C33A cells using western blot analysis. (H) E6 siRNA was transfected into C33A-pEGFP-N1-16E6 and C33A-pEGFP-N1 cells. qPCR was used to detect the level of miR-92 expression (^*^P<0.05). miR-92, microRNA-92; HPV, human papillomavirus; qPCR, quantitative PCR; NC, negative control.

In order to further investigate the role of miR-92 in cervical cancer, miR-92 expression levels were assessed in HPV16-positive and HPV16-negative cervical cancer tissues. An immunohistochemical assay was used to confirm the HPV16-E6-positive nature of 34 cervical cancer tissues, 23 of which were positive for HPV16 E6 (Fig. 2C and D). qPCR revealed that miR-92 expression was 3.56-fold higher (Fig. 1B) in the HPV16-positive cervical cancer tissues compared with the HPV16-negative tissues. The C33A and SiHa cervical cancer cell lines were then analyzed. miR-92 expression was 3.79-fold higher in the SiHa cells than in the C33A cells (Fig. 2A).

**Figure 3.**
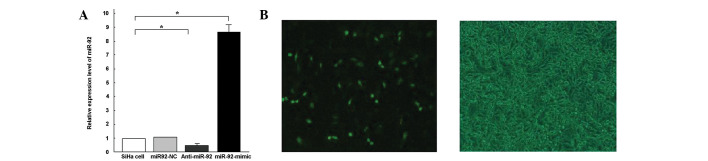
miR-92-mimic and anti-miR-92 were transfected into SiHa cells in order to alter the miR-92 levels. (A) miR-92 mimic, anti-miR-92 and NC were transfected into SiHa cells. The cells were collected and miR-92 expression was assessed (^*^P<0.05). (B) Microscopic images of cells following transfection (magnification, ×400). Following 6 h of transfection, fluorescence was used to detect E6 transfection efficiency. C33A-pEGFP-N1-E6 cells under fluorescence microscopy (left) andC33A-pEGFP-N1-E6 cells cells under light microscopy (right). miR-92, microRNA-92; NC, negative control.

The overexpression of miR-92 in the HPV16-positive cervical cancer tissues and SiHa cells prompted an investigation into the possible roles that miR-92 may play in tumorigenesis. E6 siRNA was transfected into the SiHa cells. qPCR revealed that the transfection with E6 siRNA specifically knocked down E6 expression (P<0.05; Fig. 2B). miR-92 expression was observed to be downregulated following the transfection with E6 siRNA (Fig. 2E). The pEGFP-N1-16E6 plasmid was transfected into the C33A cells to increase HPV16 E6 expression, and E6 protein expression was detected using a western blot analysis (Fig. 2G). At 6 h post-transfection, the upregulation of E6 expression was confirmed using a fluorescence microscope (Fig. 3B). The expression of miR-92 increased 5.74-fold following the transfection of pEGFP-N1-16E6 into the C33A cells (P<0.05; Fig. 2F). When E6 siRNA was transfected into the C33A-pEGFP-N1-16E6 cells, the E6 protein was blocked (Fig. 2G). The expression of miR-92 was downregulated following transfection with the E6 siRNA (P<0.05; Fig. 2H). From these results, it was concluded that the overexpression of miR-92 may be partially caused by HPV16 infection.

### Effects of miR-92 on SCC cell proliferation in vitro

**Figure 4.**
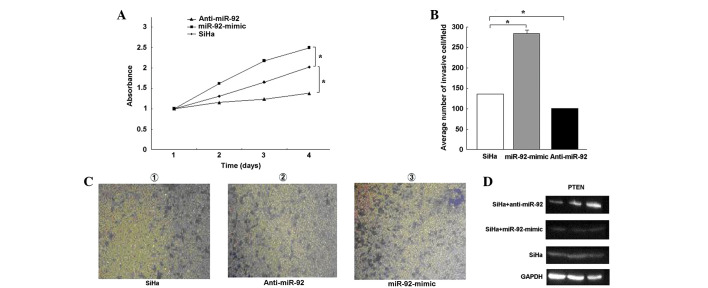
miR-92 was identified to affect the proliferative and invasive activity of SiHa cells and the expression of PTEN. (A) A CCK-8 assay was used to detect the proliferation of miR-92 on SiHa cells and the OD was determined every 24 h. (B and C) A Transwell migration assay was used to detect the invasive activity of the transfected SiHa cells (^*^P<0.05) The number of cells traversing the slide was determined by averaging five random fields (magnification, ×200) (D) Western blot analysis was used to detect PTEN protein expression when miR-92-mimic and anti-miR-92 were transfected to SiHa cells. miR-92, microRNA-92, PTEN, phosphatase and tensin homologue; CCK, cell counting kit; OD, optical density.

Since cell proliferation facilitates the development of malignancy, the present study evaluated whether miR-92 contributed to SCC cell survival. Following 6 h of transfection, miR-92 expression was detected using qPCR. miR-92 expression levels were shown to be 8.67-fold higher in the SiHa-miR-92 mimic cells than in the SiHa cells (Fig. 3A). Following 48 h of transfection, a CCK-8 assay (Fig. 4A) demonstrated that cell proliferation was significantly increased upon transfection with the miR-92-mimic and decreased upon transfection with anti-miR-92 (P<0.05), but not upon transfection with the unrelated NC. These findings indicate that miR-92 is involved in the proliferation of SCC.

### Effects of miR-92 on cell migration

The Transwell method was used to determine the effects of miR-92 on cell migration. The invasive activity of the SiHa cells increased following transfection with the miR-92-mimic. Use of the miR-92-mimic group increased the number of invasive cells by ∼2-fold compared with the miR-92-NC group (Fig. 4B and C). These results indicate that miR-92 increases proliferation and invasion in cervical cancer cells.

### Effects of miR-92-knockdown on caspase-3-dependent apoptosis in SiHa cells

**Figure 5.**
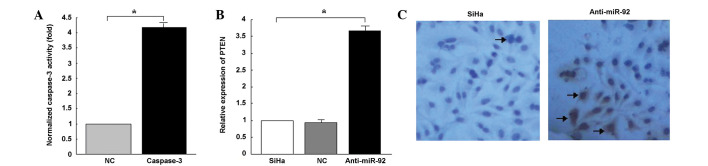
Effects of miR-92 expression on apoptosis among SiHa cells. (A) Relative caspase-3 activity in control or miR-92 inhibitor-transfected SiHa cells. (B) PCR was performed to detect the expression of PTEN mRNA in SiHa cells transfected with control and anti-miR-92. (C) TUNEL assay was performed to detect apoptosis of SiHa cells transfected with control or anti-miR-92 (arrows, apoptotic nuclei in SiHa cells; magnification, ×400). All experiments were performed in triplicate. miR-92, microRNA-92; PTEN, phosphatase and tensin homologue; TUNEL, terminal deoxynucleotidyl transferase-mediated dUTP nick end labeling; NC, negative control.

To further analyze the possible mechanisms underlying the increased sensitivity of the SiHa cells caused by anti-miR-92, the rate of apoptosis at 48 h post-transfection was detected in the SiHa cells that were transfected with anti-miR-92 and NC. A TUNEL assay indicated a significant increase in the number of apoptotic nuclei in the SiHa cells that were transfected with anti-miR-92 compared with those that were transfected with NC (Fig. 5C). Caspase-3 activity was shown to have increased 4.18-fold in the SiHa cells that were transfected with anti-miR-92 compared with the control cells (P<0.05; Fig. 5A). The downregulation of miR-92 may lead to an increase in the apoptosis of SiHa cells, which correlates with the activation of caspase-3.

### Effects of miR-92 on the regulation of PTEN protein expression in SiHa cells

PTEN protein expression is significantly downregulated in cervical cancer. miR-92 has been shown to post-transcriptionally inhibit PTEN expression in various types of human cancer cells. However, the correlation between PTEN and miR-92 in cervical cancer remains unknown. To determine the effects of miR-92 on PTEN protein expression in cervical cancer, anti-miR-92 and the miR-92 mimic were transfected into the SiHa cells. The effects of miR-92 on the endogenous expression of PTEN were then examined. qPCR demonstrated that the inhibition of endogenous miR-92 by anti-miR-92 resulted in an upregulation of PTEN mRNA (Fig. 5B). A western blot analysis revealed that the expression levels of the PTEN protein in the SiHa cells that were transfected with anti-miR-92 were higher than in those that were transfected with the miR-92-mimic (Fig. 4D).

### Effects of anti-miR-92 on tumorigenesis in cervical cancer xenografts

**Figure 6.**
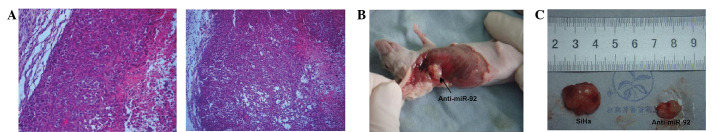
Effects of miR-92 on tumor formation in a nude mouse xenograft model. (A) Tumor xenografts with anti-miR-92 (right) and NC (left) have similar histological structures, as evaluated by HE staining (magnification, ×200). (B) and (C) Tumor specimens showing that anti-miR-92 reduced tumor formation. NC, negative control; HE, hematoxylin and eosin.

To further examine the effects of miR-92 on the *in vivo* growth of cervical carcinoma, anti-miR-92 and NC-transfected SiHa cells were independently injected subcutaneously into the two anterior flanks of the same nude mouse. The tissue structure and cell morphology of the SiHa cells that were transfected with anti-miR-92 did not differ from those that were transfected with the NC miRNAs (Fig. 6A), however, fewer tumors formed (7/10 vs. 2/10; Fig. 6B and C).

## Discussion

Evidence suggests that the aberrant expression of miRNA is involved in tumor progression, metastasis and chemoradio-resistance (17). Oncogenic miR-92 has been reported to be frequently overexpressed in a variety of human cancers (18). However, our understanding of the potential role of miR-92 in cervical cancer remains limited.

To confirm whether miR-92 expression may affect cervical cancer cells, the present study examined the expression of miR-92 in 34 cervical cancer tissue samples and 34 normal cervical tissue samples The results revealed the level of miR-92 expression in the cervical cancer tissues to be significantly higher than in the normal cervical tissues. As in previous studies conducted on other types of cancer, miR-92 was observed to be able to dramatically increase cell proliferation, inhibit apoptosis and promote cell migration in cervical cancer lines. To elucidate the mechanisms underlying the increase in chemosensitivity induced by miR-92, the effects of miR-92 on apoptosis were analyzed using SiHa cells. TUNEL assays indicated that miR-92 was able to significantly enhance apoptosis among the SiHa cells, which may be correlated with the increase in caspase-3 activity. The tumor suppressor gene, PTEN, was significantly downregulated in the SiHa-miR-92-mimic cells compared with the SiHa-NC cells. PTEN is a potential target of miR-92, which has been reported in a number of human tumors. It has been reported that the decreased expression of miR-92 may decrease cancer cell proliferation and increase the levels of PTEN, BCL2L11 and CDKN1A expression (19). Therefore, the present data indicate that miR-92 plays a number of roles in the development of cervical cancer.

HPVs are a group of small DNA tumor viruses of ∼55 nm in diameter. The DNA molecule of these viruses contains early ORFs, E1, E2, E4, E5, E6 and E7 and late ORFs, L1 and L2. The proteins that are coded by the E6 ORFs of high-risk HPVs are small nuclear proteins with transforming activities. HPV regulates the expression of numerous cellular miRNAs through the HPV E6 protein. This regulation has been shown in the reduced expression of miR-34a, miR-106b/93/25 and miR-23b through the use of E6 (20–25). High-risk E6 is able to interact with several dozen, or even hundreds of cellular factors (26–28). These interactions may lead to an increase or decrease in the expression of cellular miRNAs. In the present study, plasmid pEGFP-N1-16E6 was stably transfected into C33A cells in order to enhance E6 expression. E6 siRNA was also transfected into C33A and SiHa cells to block E6 expression. qPCR was used to detect miR-92 expression. The results suggested that the expression of miR-92 was upregulated in C33A-pEGFP-N1-16E6 cells and downregulated in HPV16 E6-knockdown cells. This indicated that HPV16 infection induces carcinogenesis, most likely by altering the expression of specific miRNAs, including miR-92. However, miR-92 may be the superior biomarker due to its broad impact on several targets and pathways involved in cervical cancer. Elucidating the role of miR-92 requires further research with regard to the number of tumor vessels that are involved in cervical disease.

In conclusion, miR-92 is overexpressed in SCC tissues and cervical cancer cell lines. Increases or decreases in the expression of miR-92 may alter multiple biological processes in human cervical cancer cells, including proliferation, apoptosis and migration, most likely through the regulation of the PTEN protein. HPV16 E6 is able to increase miR-92 expression in SiHa- and C33A-pEGFP-N1-16E6 cells. The identification of oncogenic miR-92 may serve as a biomarker for SCC. HPV may be necessary for the prevention of SCC.
